# Individual and Synergistic Effects of Tea Tree Oil and Neem Extract on Candida albicans Adhesion to Denture Soft Liner

**DOI:** 10.7759/cureus.27869

**Published:** 2022-08-10

**Authors:** Ashika Singhania, Seema Sathe, Rajul Ranka, Surekha Godbole

**Affiliations:** 1 Prosthodontics, Sharad Pawar Dental College, Datta Meghe Institute of Medical Sciences, Wardha, IND; 2 Cardiovascular Medicine, Houston Methodist Hospital, Houston, USA

**Keywords:** sabourauds agar, denture stomatitis, soft liner, azadirachta indica, melaleuca alternifolia, candida albicans

## Abstract

Background

Inflammation is present in denture stomatitis. Denture stomatitis's etiology is complex, but there is evidence that it is brought on by *Candida albicans* growing in biofilms on its surface.

Objective

This study aimed to examine and assess the effectiveness of several herbal products, such as tea tree oil and neem extracts, on *Candida albicans* adhesion to denture soft liners.

Method

Each wall of the tissue culture plate was filled with 30 acrylic blocks lined with soft liners, followed by the addition of 0.1 ml of the standardized *Candida albicans* suspension, which was then left to incubate for 48 hours. Each specimen was placed in a disinfectant solution for 10 minutes. A colony was counted after 0.1ml of the solution was plated on a sabouraud dextrose agar (SDA) plate and cultured for 72 hours. Data were compared using a one-way analysis of variance (ANOVA) test.

Results

The mean colony forming units (CFU) per ml for combined tea tree oil and neem extract was least (0.40), followed by tea tree oil (2.30), followed by neem extract (30.33). The treated blocks were effective in reducing the growth of *Candida albicans*.

Conclusion

Combining tea tree oil and neem extract significantly reduced the growth of *Candida albicans, *suggesting a new form of intraoral effective antifungal treatment.

## Introduction

Due to higher life expectancies, elderly populations are more prevalent in developed nations. This increase in the senior population could result in more people needing removable dentures. Due to limited exposure to saliva's purifying effects, the environment under a denture is favorable for the growth of *Candida albicans* (*C. albicans*) [1].

A typical commensal microorganism found in the human oral cavity is *C. albicans*. The posterior part of the tongue and other oral sites are where it is most prevalent, and the film that covers the tooth surfaces is where it gets secondarily colonized [1]. A common benign condition known as Candida-associated denture stomatitis (DS) regularly affects people who wear dentures. It's a chronic inflammatory disorder that causes erythema in the oral tissues that support a detachable prosthesis [2]. Oral candidiasis has been treated with a variety of potent antifungal medications, both topically and internally. Amphotericin B and nystatin are two popular topical antifungal medications, while fluconazole and ketoconazole are available as systemic antifungal medications. Although systemic dosage forms may be successful against mucosal infections, they do not provide a cure for the tissue surface of dentures that are infected with *Candida* [3].

Numerous medicinal plants have been utilized as anti-fungal agents, including tea tree oil, aqueous neem extract, and eucalyptus globulus essential oil [4,5]. The tea tree is commonly known as *Melaleuca alternifolia (M. alternifolia)*. It exhibits a broad spectrum of antimicrobial activity and is effective against other pathogenic yeasts and gram-positive and negative bacteria [6]. Neem is a medicinal tree that is scientifically referred to as *Azadirachta indica (A. indica)*. *A. indica extracts* can effectively suppress several human fungi, including *Geotrichum, Trichosporon, Candida, Epidermophyton, Trichophyton, and Microsporum* [7]. The purpose of this study was to compare the individual and synergistic effects of *M. alternifolia* and *A. indica* on the growth of *C. albicans*.

## Materials and methods

**Figure 1:**
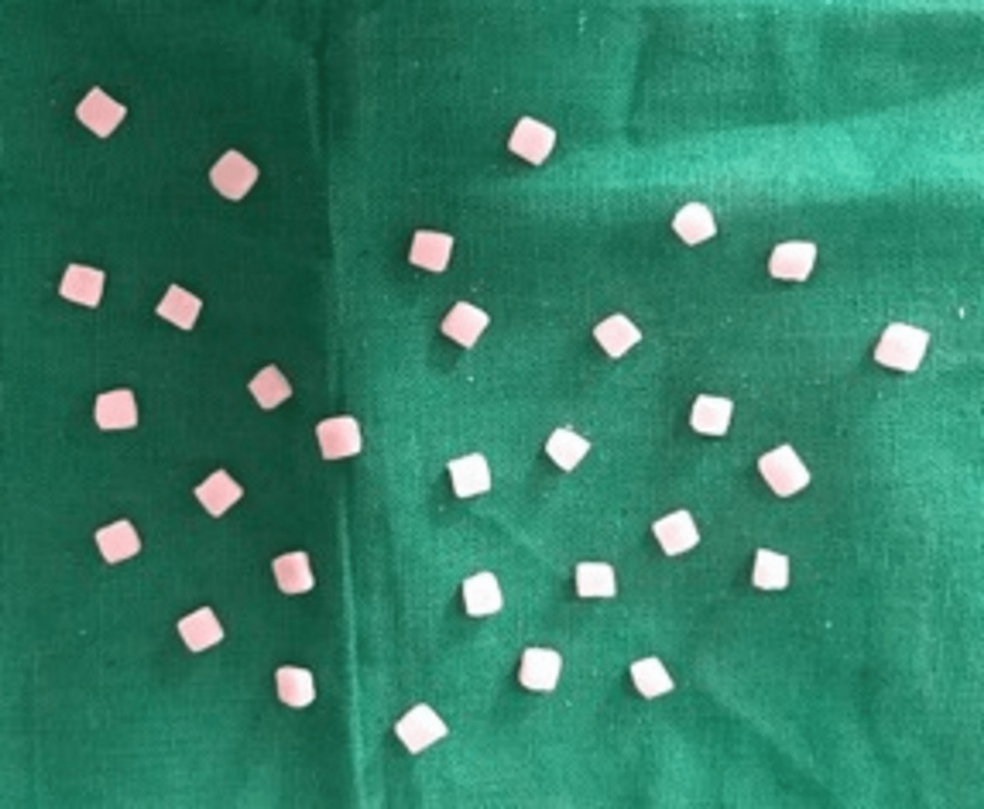
Heat cure acrylic samples

A total of 90 samples were made out of modeling wax using a laser-cut mold with dimensions of 4mm X 4mm X 2mm, and then they were acrylic into heat-cured acrylic resin samples (Figure 1).

**Figure 2:**
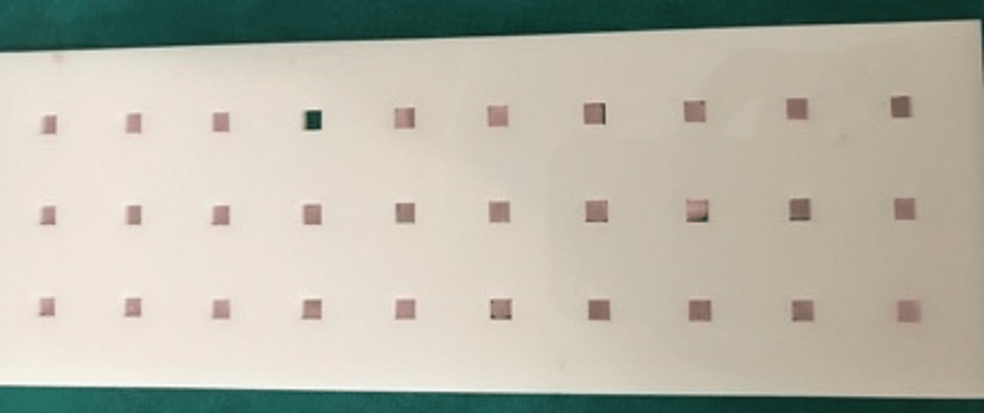
Samples lined with soft liner

A soft denture relines material (GC Corporation, Tokyo, Japan) was applied to each block at a thickness of about 2 mm to provide an even layer when placed in the mold of dimensions 4mmX4mmX4mm after being cleaned by buffing with pumice and then with running water. The surplus material was then cut off using a BP blade after it had been allowed to sit for 10 minutes to acquire 90 samples (Figure 2).

The study consisted of three groups of 30 samples each; group 1 samples immersed in tea tree oil, group 2 samples immersed in alcohol-based neem extract, and group 3 samples immersed in a combined tea tree oil-neem extract. 

**Figure 3:**
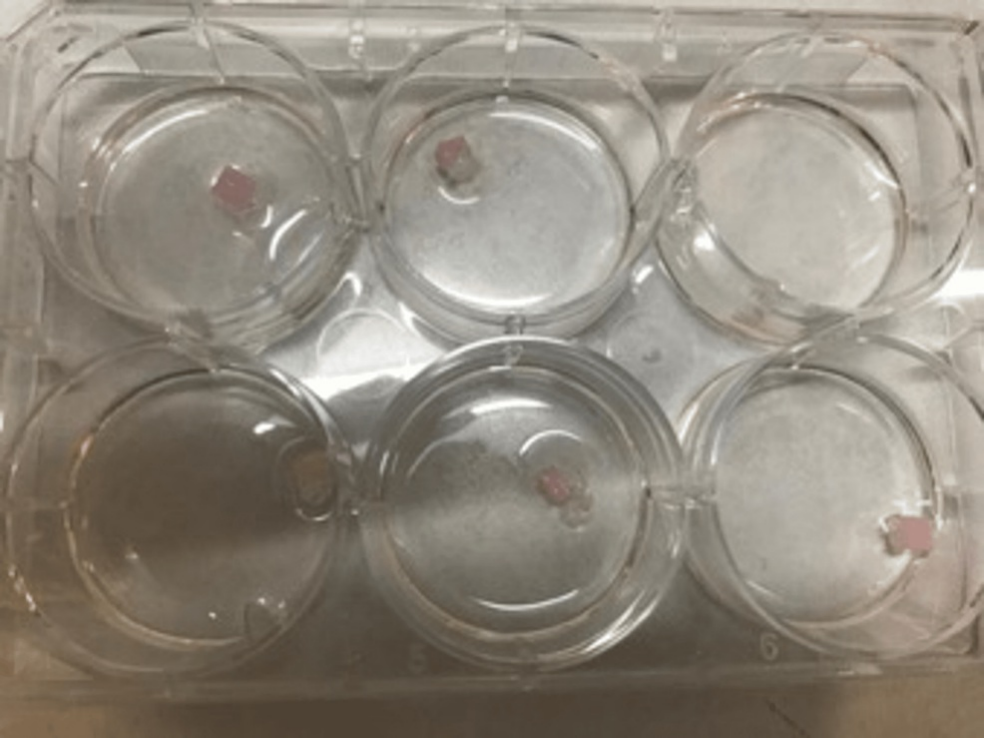
Acrylic specimen placed in well of tissue culture plate

A 10 ml suspension of the growth was prepared in a test tube by adding *Candida* growth from SDA medium by using a bacteriological wire loop to sterile saline solution and placed in a cyclomixer for 60 seconds. The suspension was then standardized to contain 1.5X10^8^ colony forming units using the 0.5 McFarland turbidity standard by matching the turbidity. One (soft lined acrylic block) specimen was placed in each well of the tissue culture plate. Using a 2 ml disposable syringe, 1.5 ml of Sabouraud's dextrose broth was added to each well, and 0.1 ml of *Candida albicans* standardized suspension was added with the help of a micropipette to each well. The plates were sealed and incubated in the incubator for 48 hours at 37° C (Figure 3).

**Figure 4:**
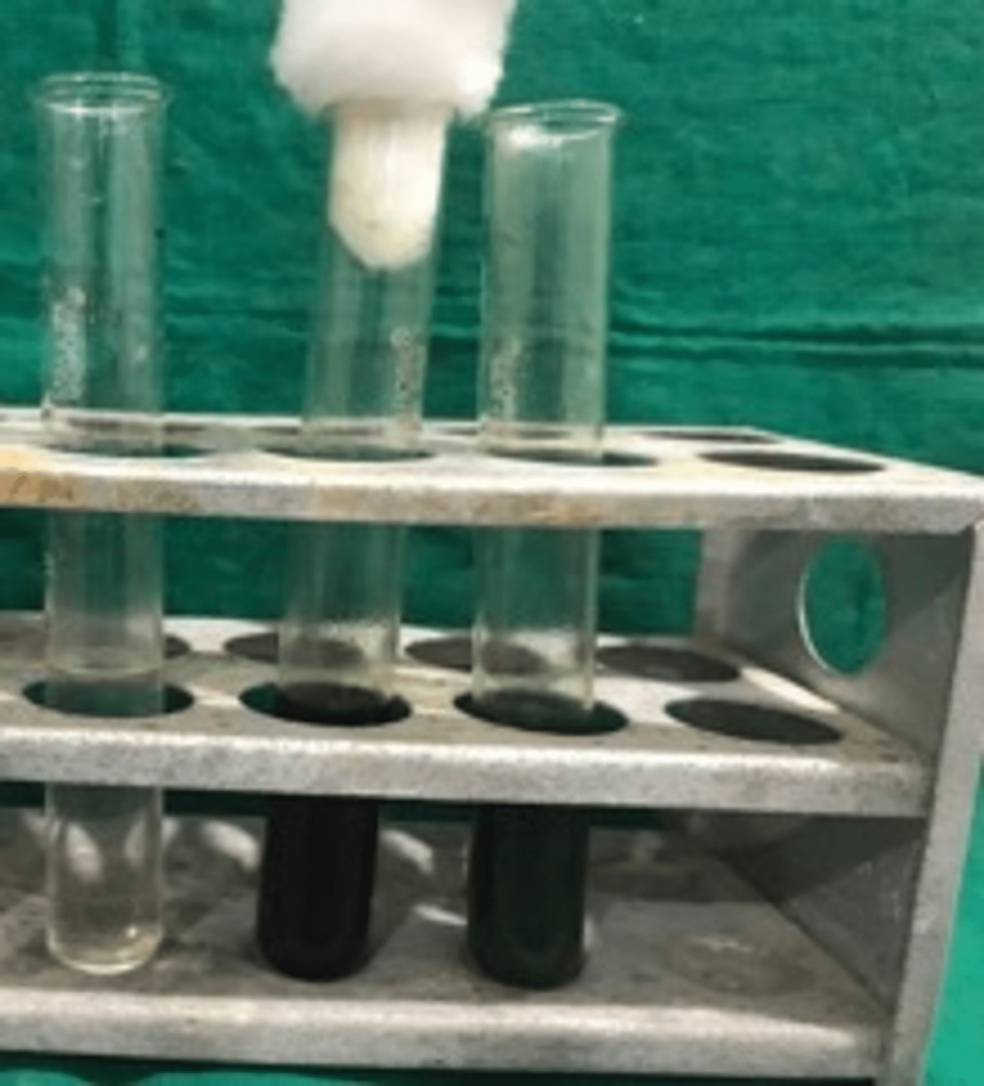
10ml of each disinfectant

Ten ml of each disinfectant solution were taken in 20 ml test tubes using a pipette, and 30 specimens were immersed in the corresponding solution for 10 minutes according to the group mentioned (Figure 4).

Then each specimen was transferred to tubes containing 1 ml of sterile physiological solution, and the tubes were agitated in a cyclomixer for 60 seconds to disperse adhered cells. Direct subculture from the suspension may result in confluent growth of *Candida albicans*, and colony counting may be impossible, so to get a proper colony count, the initial suspension was diluted 10, 100, and 1,000 times in the physiological solution.

**Figure 5:**
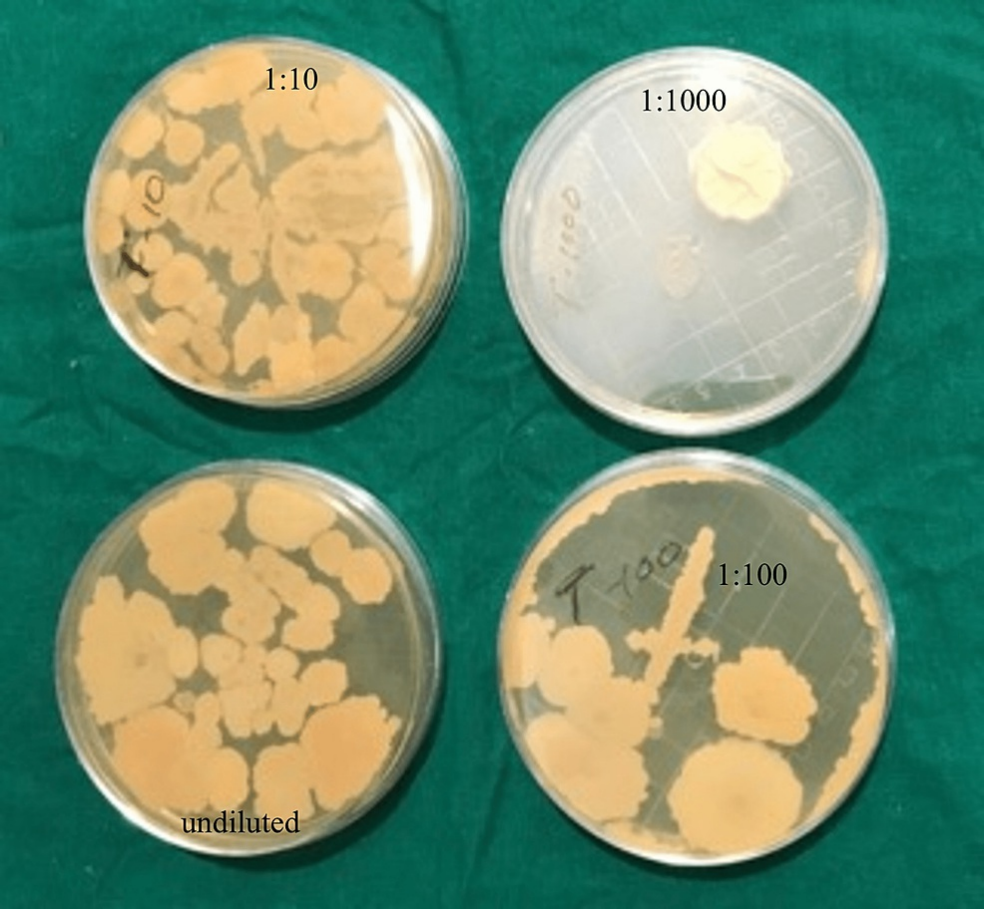
Colonies on normal, 10, 100, 1000 dilution of tea tree oil

**Figure 6:**
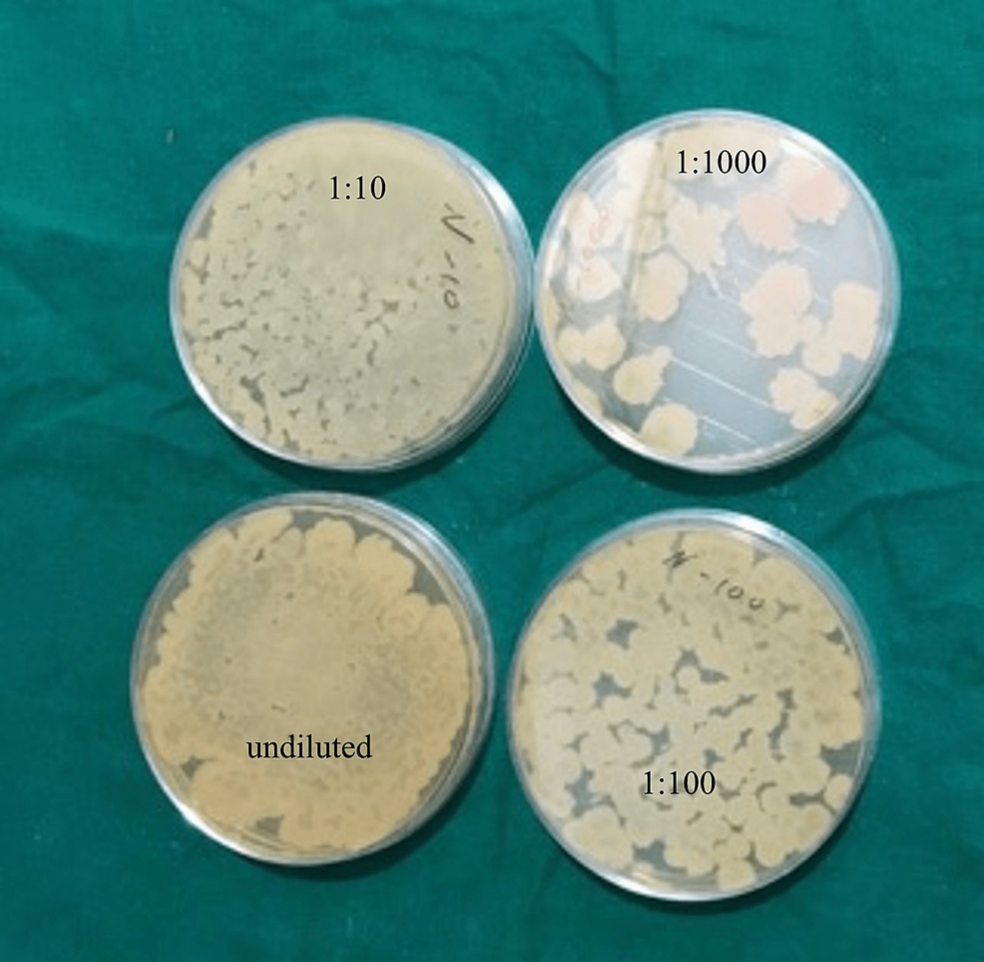
Colonies on normal, 10, 100, 1000 dilution of neem extract

**Figure 7:**
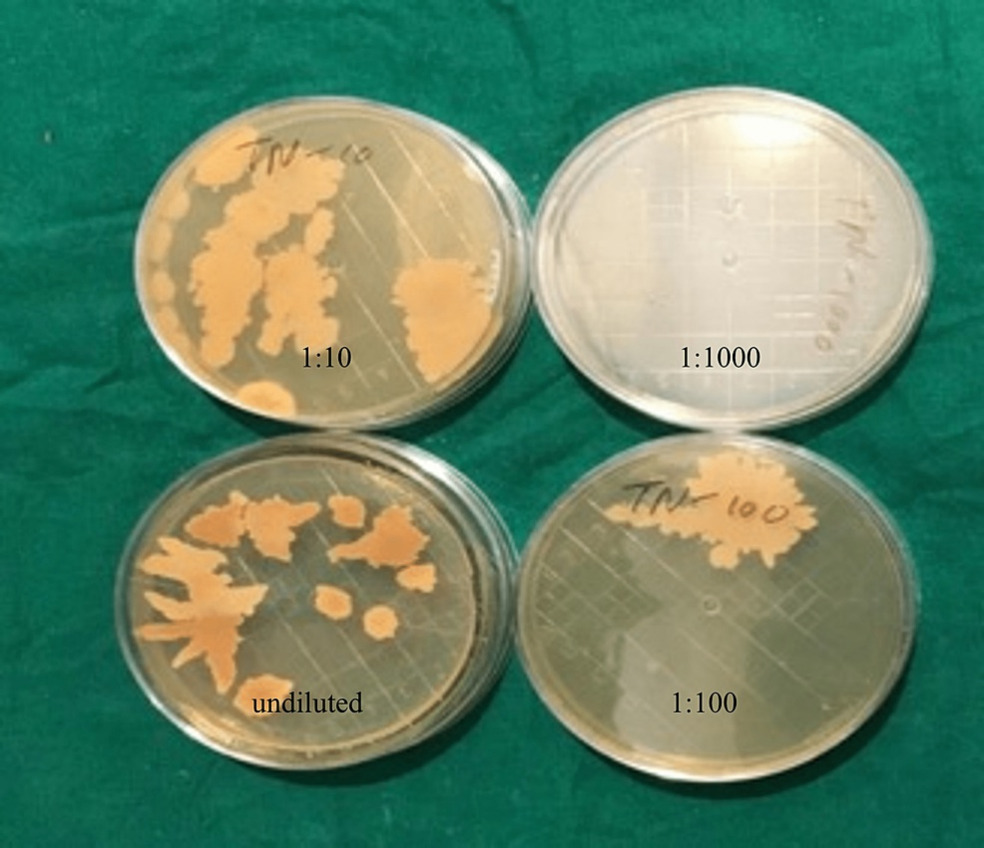
Colonies on normal, 10, 100, 1000 dilution of combined tea tree oil and neem extract Colony count: dilution factor/culture plate volume

0.1 ml of each suspension was taken using a micropipette and plated on a Sabouraud dextrose agar plate. A total of four plates for each sample were plated, i.e., undiluted solution, 1:10 dilution, 1:100 dilution, and 1:1000 dilution. After 72 hrs. of incubation at 37° C, the numbers of colonies in each plate of each disinfectant, i.e., undiluted, 1:10, 1:100, and 1:1000, were counted using a digital colony counter, and the number of colonies forming units/ml was calculated (Figures 5, 6, 7).

## Results

One-way ANOVA test was used to analyze the result using IBM Corp. Released 2017. IBM SPSS Statistics for Windows, Version 25.0. Armonk, NY: IBM Corp. On dilution with neem extract colony count was (30.33 ± 4.99) (p-value 0.001), and then the colony count decreases with tea tree oil (2.30±1.18) (p-value 0.001). The least colony count was seen with combined tea tree oil and neem extract (0.40± 0.77) (p-value 0.001). Post hoc analysis showed a comparison of the tea tree oil group with the neem extract group was statistically significant (p-value 0.001), whereas the comparison of the tea tree oil group with the tea tree oil group and neem extract group was statistically not significant (p-value 0.396). Statistically significant result was seen when the neem extract group was compared with the tea tree oil and neem extract group (p-value 0.001) (Table 1).

**Table 1:** Comparison between tea tree oil, neem extract and combined tea tree oil and neem extract

		N	Mean	Std. Deviation	F	p-value	
Tea tree oil		30	2.30	1.18			
Neem extract		30	30.33	4.99			
Tea tree oil and neem extract		30	0.40	0.77			
Post-hoc analysis using LSD							
GROUP	GROUP					p-value	
Tea tree oil	Neem extract					0.001*	
	Tea tree oil and Neem extract					0.396	
Neem extract	Tea tree oil and Neem extract					0.001*	

## Discussion

Over time, there has been a gradual rise in the aged population. As a result, elderly people are now a group of people who require particular care and dental care models that result in preventive, rehabilitative, curative, and humanized assistant levels [8]. In addition to improving mastication [9], the treatment objectives for edentulous patients using artificial prostheses include comfort, aesthetics, enhanced speech, and helping them regain their confidence. The material used for making dentures is either metallic or non-metallic. The majority of complete dentures are fabricated using acrylic resin. The merits of using acrylic resin are its ease of manipulation and cost efficiency. While the disadvantage is that they present an additional hard porous surface with a high rate of adsorption, allowing local microtrauma, which causes inflammatory reactions and supports fungal growth, as well as adherence and plaque development [10].

Denture-associated stomatitis, also known as chronic atrophic candidiasis or erythematous candidiasis, is the most prevalent type of oral candida infection in which fungal growth is seen. It significantly affects denture users and can be treated by various preventive and therapeutic methods like denture cleaning, removal of dentures at night and antifungal drugs [10]. The negative effects of antifungal medications, despite their effectiveness, are diverse and include toxicity, drug-drug interactions, recurrence, the development of fungal resistance, and high cost. So natural products came into being since resistance is seen by using various antifungal, antibacterial, antioxidant, and anti-inflammatory effects [11]. 

In this study, when the acrylic blocks lined with soft liners were immersed in Group 1 (Tea tree oil) for 10 minutes, the colony forming units observed was extremely low, showing only 2.30+/-1.18 (Table 1) with a p-value of 0.001. At a dosage of 0.25 percent, TTO can block the development of germ tubes and mycelial conversion, slowing the growth of *C. albicans* [9]. TTO contains terpenine-4-ol, which increases yeast cell permeability by rupturing the barrier of microbial membrane structure, increases membrane fluidity, and prevents the medium from becoming too acidic, which prevents the fungi from breathing [12-14]. This study was supported by Pachava KR in 2015, where the mean of colony forming units in tea tree oil treated acrylic discs were checked after one day, 30 days, and 60 days and the colonization was lower in tea tree oil treated discs with a p-value of 0.001 [15]. Vankadara SK in 2017 conducted a study wherein the colonization and inhibition of *Candida albicans* is checked on commercially available denture lining materials by subjecting them to various concentrations and doses of tea tree oil, and a lesser zone of inhibition was seen with a 2 ml volume and 40% concentration of tea tree oil on GC soft liner [16]. G Mahalakshmi in 2020 conducted a study wherein tea tree oil was added to denture soft liner, and the growth of *Candida albicans *significantly reduced to 1.8 × 10^6^, 2.6 × 10^6^, 32.9 × 10^6^ after 1, 30, and 60 days [17]. P Kumar, in her 2020 study, compared the antifungal activity of the MIC of the three oils by measuring the mean zone of inhibition and reached a conclusion that for the treatment of denture stomatitis, the antimycotic properties of *M. alternifolia*, *C. nucifera*, and *A. indica* combined with the Visco-gel tissue conditioner can be employed [18].

When the acrylic blocks lined with soft liners were immersed in Group 2 (neem extract) for 10 minutes, the colony-forming units were reduced to 30+/-4.99 with a p-value of 0.001, which proves its antifungal property (Table 1). The most active phytochemical in neem is azadirachtin [7]. Neem leaves may have an anti-adhesive action that prevents the microorganism's initial adherence and has an impact on biofilm formation and cell surface hydrophobicity, which lessens *C. albicans *colonization [5,19]. Due to the lower concentration of bioactive components in aqueous neem extract, the latter has a stronger fungicidal impact [5]. In her study in 2011, Aarati N proved that alcoholic leaf extract was found to be more effective than aqueous neem extract against *S. mutans, Enterococcus faecalis*, and *C. albicans* [20]. Kumar SM in 2018 conducted a study where the inhibitory effect of *Candida albicans* was checked on denture soft liner by addition of garlic and neem and both garlic and neem showed positive effects and helped reduce the growth of *C. albicans *[21]. G Krishnaveni conducted a similar study in 2021 where soft-lined acrylic blocks were immersed in disinfectant solutions such as distilled water, 100% tea tree oil, 100% neem extract, 2% chlorhexidine, and 0.5% sodium hypochlorite and observed that tea tree oil and neem extract had the highest inhibitory effect on the growth of *Candida albicans* with every dilution; however, the efficacy of neem extract reduced with an increase in dilution [22]. AlHamdan E conducted a study in 2022 where soft-lined removable acrylic complete dentures were fabricated and immersed in group 1: 5 µm of Rose Bengal (RB), group 2: Neem extract, group 3: Tea tree oil (TTO), and group 4: 0.12% CHX solutions, respectively, and concluded that the count of *C. albicans* unveiled a significant plunge with CHX, TTO, and neem extract except RB (p<0.05) [23].

In this study, synergistic effects were tested between the two components, which had never been done before. When the acrylic blocks lined with soft liners were immersed in Group 3 (combined TTO-neem) for 10 minutes, the colony forming units decreased, and there were no colonies seen after a 1:1000 dilution, and the p-value seen was 0.001 (Table 1). The absence of colonies with the use of combined TTO-neem as a disinfectant proves its fungicidal effect and its enhanced efficacy compared with other disinfectants. This proves that the synergism between TTO and neem shows a fungicidal property. 

According to the literature, the synergistic approach of essential oils appears to be more advantageous and effective when compared to their solo application. This combination may be between either two or more essential oils, or it can be between antibiotic and essential oil. Rathod T in 2017 proved the synergistic action of Fluconazole with both the essential oils, *A. indica* and* Citrus reticulate* and showed antifungal properties against *C. albicans* [24].

In the present study, we checked the growth of only *Candida albicans* because, amongst the fungi seen in the oral cavity, about 80% of the microorganisms that are recoverable from the oral mucosa of denture wearers consist of *Candida albicans* [25]. The most common form of oral *candida* infection, which affects a great number of denture wearers, is denture-associated stomatitis, also known as chronic atrophic candidiasis or erythematous candidiasis [26]. The various treatment modalities for denture-induced stomatitis include relining with tissue conditioners, maintaining good oral hygiene, removing the dentures at night, and use of various antifungal substances like sodium hypochlorite, chlorhexidine, fluconazole, and amphotericin B [27]. Due to the above-mentioned disadvantages of anti-fungal medicines, in our study, we compared the anti-fungal efficacy of different natural products, which have relatively fewer side effects. 

In light of the study's encouraging findings, natural products can be employed as less side-effect-prone replacements for chemical medications to treat *C. albicans*. These findings urge additional research into both the numerous synergistic interactions between plant oils or plant oils and antifungal medications as well as the effectiveness of various other plant oils against *C. albicans*.

The study had the following limitations: it only tested *Candida albicans* as the microorganism causing denture stomatitis. The effect of other microflora on denture stomatitis was not evaluated. Further in vivo studies on a large scale should be conducted to confirm the findings of the efficacy of disinfectants.

## Conclusions

As they exhibit resistance to all antifungal medications, the resistant strains of *C. albicans* have grown to be a significant source of health risks. The medicinal and herbal effects of plants could offer a possible antifungal lead against *C. albicans-*resistant strains. For the production of herbal mouthwashes and denture disinfectants, standardized formulations of these medicinal plants with antibacterial activity are needed. Hence, within the limitations of the study, the following conclusions were drawn: that the colony count and colony forming units were least in combined tea tree oil-neem with each dilution, and there were no colonies seen on combined tea tree oil-neem when compared with other disinfectants. 
